# Transcription factor Foxp1 is essential for the induction of choroidal neovascularization

**DOI:** 10.1186/s40662-022-00281-7

**Published:** 2022-03-06

**Authors:** Meifang Yan, Junjian Li, Li Yan, Xue Li, Jie-Guang Chen

**Affiliations:** 1 School of Ophthalmology and Optometry and Eye Hospital, Wenzhou Medical University, State Key Laboratory of Optometry, Ophthalmology and Vision Science, and Zhejiang Provincial Key Laboratory of Optometry and Ophthalmology, 270 Xueyuan Road, Wenzhou, Zhejiang 325027 People’s Republic of China; 2 Joint Shantou International Eye Center (JSIEC) of Shantou University and the Chinese University of Hong Kong, Shantou, Guangdong 515000 People’s Republic of China

**Keywords:** Foxp1, Choroidal neovascularization, Angiogenesis, Laser photocoagulation, Age-related macular degeneration

## Abstract

**Background:**

The exudative form of age-related macular degeneration (AMD) is characterized by abnormal blood vessel growth, which is stimulated by vascular endothelial growth factor (VEGF) released from retinal pigment epithelium (RPE). The angiogenic behaviors of vascular endothelial cells in vitro depend on forkhead box protein P1 (Foxp1), a transcription repressor widely expressed in human and murine tissues during development. In this study, we aimed to determine whether loss of Foxp1 affects laser-induced choroidal neovascularization (CNV) in mouse.

**Methods:**

Eye-selective deletion of Foxp1 was obtained by crossing Foxp1^flox/flox^ with Six3-Cre mice. Laser photocoagulation was delivered to six- to eight-week-old mice to induce CNV. The expression of Foxp1 and Cre was determined by immunofluorescence in cryostat sections of the eyes. Fundus fluorescein angiography (FFA), optical coherence tomography (OCT), and B4 isolectin staining were applied to analyze the leakage, bulge height, and area of CNV lesions, respectively. RPE-choroid tissues were isolated for the determination of VEGF and pigment epithelium derived factor (PEDF) by Western blotting.

**Results:**

Foxp1 was expressed in retinal ganglion cells, RPE, and the choroidal endothelial cells. Laser photocoagulation increased the number of Foxp1^+^-endothelial cells and induced CNV. Six3-Cre reduced Foxp1 expression in RPE but not the endothelium, leading to a lower level of VEGF in the RPE-choroid. Foxp1 knockout inhibited pathological angiogenesis and vascular leakage of the laser-induced CNV lesions.

**Conclusions:**

Foxp1 regulates the expression of VEGF in the RPE, and inhibition of Foxp1 could potentially be a novel strategy for the prevention and therapy of neovascularization related to AMD.

## Background

Age-related macular degeneration (AMD) is a complex, multifactorial disease, and a primary cause of blindness among the elderly. Approximately 10% of AMD patients suffer from the exudative or wet form of AMD that is characterized by choroidal neovascularization (CNV). The newly-formed blood vessels from the choroid invade the retina through fractures in Bruch’s membrane, the extracellular matrix between the choroid and the retinal pigment epithelium (RPE). The immature blood vessels leak fluid below and within the retina, causing edema in the eye and central vision loss. The vision-threatening vasculopathy is associated with an abnormal increase of vascular endothelial growth factor (VEGF) [1], a potent pro-angiogenic molecule in the choroid. In animal studies, increased expression of VEGF in the RPE can lead to CNV [2]. Currently, agents that effectively inhibit VEGF become the therapeutic basis for neovascular AMD [3, 4]. However, VEGF helps the maintenance and survival of RPE, and long-term anti-VEGF treatment may have severe side effects and result in geographic atrophy of the ocular tissues [5, 6]. Thus, it remains a challenge to search for new therapeutics based on a better understanding of the genetic mechanisms of neovascular AMD and the molecular regulation of VEGF.

The incidence and progression of AMD increase significantly with age. Multiple conditions accelerate the development of AMD, including dysregulated complement system, oxidative stress, impaired autophagy clearance, and inflammation of endothelial cells [3, 7, 8]. Transcription factor forkhead box protein P1 (Foxp1) is a key regulator of endothelial cell inflammation and plays an important role in heart development [9, 10]. Targeted deletion of Foxp1 in vascular endothelial cells results in increased atherosclerosis in mice by modulating NLRP3 inflammasome activation [11]. In ischemic hindlimb, Foxp1 is highly expressed in endothelial cells at the site of neovascularization. Silencing Foxp1 in vitro results in semaphorin 5B-mediated inhibition of endothelial cell proliferation, migration, and sprouting [12]. These observations show that Foxp1 is essential for angiogenesis in endothelial cells. However, it has not been examined whether this transcription factor regulates the choroidal VEGF, a critical player in CNV.

Foxp1 has been linked to various cognitive disorders. Foxp1-specific deletions, mutations, and chromosomal breakpoints interrupting the gene have been reported in autistic patients with intellectual disabilities and or speech and language deficits [13, 14]. The loss of Foxp1 in autistic patients frequently coincide with ophthalmological abnormalities [15], and autism is unusually common among blind people [13, 16]. As AMD is the primary cause of blindness among the elderly, this study aimed to investigate the roles of Foxp1 in CNV by eye-selective deletion of Foxp1. We crossed Foxp1^flox/flox^ with Six3-Cre mice. Foxp1, but not Six3-Cre, was expressed in choroidal endothelial cells. Six3-Cre was expressed in the RPE cells, and Foxp1 was reduced by the knockout, leading to a lower level of VEGF in the RPE and choroid. Foxp1 knockout (KO) inhibited pathological angiogenesis and vascular leakage of the laser-induced CNV lesion. The results suggest that inhibition of Foxp1 could potentially be a novel strategy for the prevention and therapy of neovascularization related to AMD.

## Methods

### Animals

Six- to eight-week-old C57BL/6 J mice (Shanghai Jiesijie Laboratory Animal Co., Ltd.), Six3-Cre mice, EGFP-L10a, and Foxp1^flox/flox^ mice (Jackson Lab) used in this study were housed under a 12 h light/dark cycle and given ad libitum access to food and water. All animal experiments were carried out according to the ARVO Statement for the Use of Animals in Ophthalmic and Vision Research. The study was approved by the Laboratory Animal Ethics Committee of Wenzhou Medical University (ID Number: xmsq2021-0163). To knockout Foxp1 in the eye, Foxp1^flox/flox^ mice were crossed with the Six3-Cre to generate heterozygous F1 progeny, which was backcrossed with Foxp1^flox/flox^. The F2 progenies were genotyped by polymerase chain reaction (PCR) according to the protocol provided by Jackson Laboratory, with the following primers: Foxp1^flox/flox^ Forward (5’-TGGTTCACACGAATGTTTGC-3’) and the reverse primer, (5’-GGAGTGGCTCTTCCATCTGA-3’); Six3-Cre Forward (5’-TCGATGCAACGAGTGATGAG-3’) and the reverse primer (5’-TTCGGCTATACGTAACAGGG-3’). The double deficient KO mice were identified by the presence of Cre and floxed Foxp1 in their tail DNA. The wild-type (WT) littermates, as with the experimental controls, were those that harbor the floxed allele of Foxp1 but without Cre. Mice were euthanized via intraperitoneal injection of pentobarbital (50 mg/kg).

### Laser-induced choroidal neovascularization

CNV was induced by laser photocoagulation of Bruch’s membrane using the image-guided laser system (Micron IV, Phoenix Research Labs, USA). The mice were anesthetized by intraperitoneal injection of ketamine (100 mg/kg) and xylazine (10 mg/kg) diluted in sterile 0.9% saline. The pupils were dilated using compound tropicamide eye drops (Handan Kangye Pharmaceutical Co., Ltd.). Argon laser (Merilas 532α, 532 nm wavelength, 100 mW output energy, 70 ms duration, 50 μm spot size) was used to produce four laser burns in the fundus of each eye. The burning spots were 1–2 papillary diameters away from the optic disc. We considered the occurrence of a vaporizing bubble as evidence of successful laser treatment. The eyes were coated with ofloxacin eye ointment (Shenyang Xingqi Eye Medicine Co., Ltd.) after the laser burn. The mice were put on a heating plate to help their recovery from the experiment. Eyes with excessive laser burns and severe hemorrhage lesions were excluded from the experiments [17].

### Fundus fluorescein angiography (FFA)

On day six post laser treatment, FFA was used to assess the leakage of CNV. Mice were anesthetized and injected intraperitoneally with fluorescein sodium solution (2.5% in saline, 0.1 ml). FFA images were taken by Micron IV retinal imaging system at 5 min (early phase) and 10 min (late phase) following the injection. The mean fluorescence intensity (FI) of CNV lesions was calculated using ImageJ (National Institutes of Health, Bethesda, MD) software, and the change was expressed as a percentage of the increase: [(FI_10min_-FI_5min_) ÷ FI_5min_] × 100%. CNV leakage was evaluated by scorers blinded to the experimental conditions using the following grading criteria: grade 0 (without leakage): faint or mottled fluorescence; grade 1 (questionable leakage): hyper-fluorescence lesion without a progressive increase in size or intensity; grade 2a (leaky): hyper-fluorescence with an increase in intensity but not in size; grade 2b (pathologically significant leakage): hyper-fluorescence increasing in both intensity and size [18, 19].

### Optical coherence tomography (OCT)

One day after FFA, when the eyes have recovered from the anesthesia-related opacification of the intraocular lens, a spectral domain optical coherence tomography (SD-OCT) system (Spectralis, Heidelberg Engineering, Heidelberg, Germany) was used for imaging the cross-section of the fundus in real-time. Mice were anesthetized, and the pupils were dilated. The eyes were kept moist using sodium hyaluronate eye drops (Zhejiang Jianfeng Pharmaceutical Co., Ltd.). We chose a deep-enhanced imaging model and B-scan to capture the CNV lesion images. The edges of CNV were traced by ImageJ to help calculate the thickness, defined as the height from the inner border of the RPE to the highest point of the CNV area.

### Preparation and staining of RPE-choroid-sclera complex and retina flat-mounts

The eyeballs of mice were harvested and fixed in 4% paraformaldehyde (PFA) in phosphate buffered solution (PBS) for 10 min at room temperature. The anterior segment and lens were removed, and the optic nerves were cut off. The neural retina was carefully separated from the RPE-choroid-sclera complex. RPE-choroid-sclera and the retina were post-fixed in PFA for 20 min, washed in PBS, and permeabilized in 0.5% Triton X-100 (in PBS) for 4 h. The tissues were stained for 12 h at room temperature with Alexa Fluor® 594-conjugated GSL I-B4 isolectin (1:300, I21413, Invitrogen), a marker for endothelial cells. To visualize the vascular structures, we flat-mounted RPE-choroid-sclera and the neural retina onto slides in an anti-fade mounting medium (P0126, Beyotime, China). Fluorescent images were captured by a Zeiss LSM 880 microscope.

### FITC-dextran perfusion and melanin bleaching

Fluorescein Isothiocyanate dextran (FITC-dextran: FD2000S-1G, Sigma) was diluted in distilled water to a concentration of 50 mg/ml. Mice were anesthetized, and 0.2 ml FITC-dextran was injected into the left ventricle of the mouse. Eight minutes later, the mice were sacrificed. Eyeballs were enucleated and fixed in 4% PFA for 1 h at room temperature. After washing in PBS, the eyeballs were incubated with 2% hydrogen peroxide (H_2_O_2_) for 15 min at room temperature and then 10% H_2_O_2_ at 55 ℃ for 2.5 h to bleach the pigment [20]. The eyeballs were washed in PBS, and the anterior segment and lens were removed. The RPE-choroid-sclera complex and the neural retina were prepared and mounted on glass slides. Fluorescent images were captured by a Zeiss LSM 880 microscope.

### Immunofluorescence

To determine the expression of Foxp1 and cell marker proteins, we prepared frozen sections of the eyeballs. Eyeballs were first fixed in 4% PFA for 0.5 h, another 1.5 h after poking a hole in the cornea and dehydrated in 30% sucrose. The anterior segment was cut off, and the remaining were frozen in OCT compound (Sakura Tissue Tek, Torrance, CA) and sectioned with a cryostat (HM505E, Microm, Germany) at 10 μm. The cryosections were permeabilized and blocked in PBS containing 0.3% Triton X-100, 1% BSA, and 5% donkey serum for 2 h at room temperature. After overnight incubation at 4 ℃ with primary antibodies, the sections were washed with PBS, incubated with secondary antibodies for 2 h, and mounted on glass slides in Slowfade Gold medium (Invitrogen) that contains DAPI for nuclear staining. The primary antibodies used in this study were anti-CD31 antibody (1:300, ab7388, Abcam), anti-FOXP1 antibody (1:100, ab227649, Abcam), anti-CRE antibody (1:300, Sigma, MAB3120), anti-VEGF antibody (1:50, MA5-13182, Invitrogen), anti-pigment epithelium derived factor (PEDF) antibody (1:100, ab180711, Abcam) and anti-OTX2 antibody (1:300, AF1979, R&D Systems). In control experiments, the primary antibodies were replaced by PBS. The secondary antibodies from Jackson ImmunoResearch Laboratories include Cy3-conjugated anti-Rabbit (1:400), Alexa Fluor^TM^488 anti-Rat IgG, Alexa Fluor^TM^488 anti-Mouse IgG, Alexa Fluor^TM^488 anti-Goat IgG, Alexa Fluor^TM^647 anti-Rat IgG, Alexa Fluor^TM^647 anti-Mouse IgG, and Alexa Fluor^TM^647 anti-Goat IgG. Immunofluorescence images were captured using a confocal microscope (Zeiss LSM 880).

For detection of Cre recombinase activity, Six3-Cre transgenic mice were crossed with the EGFP-L10a reporter strain (Jackson Lab). The reporter allows the fusion protein of EGFP and ribosomal L10a to be expressed in specific cell populations in response to the presence of Cre recombinase, which removes a loxp-flanked stop cassette placed between the promoter and the fusion gene [21]. The eyeballs from the adult progenies were harvested, and the cryostat sections were prepared as above. EGFP after the Cre mediated recombination was detected by a fluorescence microscope.

### Western blot analysis

RPE-choroid tissues were isolated from Foxp1 KO and the control littermates following an established protocol [22]. Briefly, the anterior segment and neural retina were removed from the eyes. The RPE-choroid was separated from the sclera and lysed in ice-cold RIPA buffer (P0013B, Beyotime Biotech, China) containing a cocktail of protease inhibitors (5871S, Cell Signaling Technologies, USA). The protein concentration in isolation was measured by BCA Protein Assay Kit (P0010, Beyotime, China). The samples were loaded onto sodium dodecyl sulfate–polyacrylamide gels, separated by electrophoresis, transferred to polyvinylidene fluoride membranes (Merck Millipore). The membrane was blocked in TBS-T (150 mM NaCl, 10 mM TRIS–HCl pH 7.5, and 0.1% Tween 20) containing 5% (w/v) dry milk and probed separately with Mouse anti-VEGF-A (1:200, ab1316, Abcam), Rabbit anti-PEDF (1:2000, ab180711, Abcam) and Mouse anti-α-tubulin (1:4000, ab52866, Abcam) antibodies. The signals were detected with secondary antibodies and quantified using Odyssey infrared laser imaging system (LI-COR Biosciences).

### Statistical analysis

For all the measurements in this study, data were presented as mean ± standard deviation (SD) from 3 to 5 mice in each group. The student’s *t*-test was used for the comparison between two groups. As for multiple comparisons, one-way ANOVA was performed, followed by Tukey’s post-hoc multiple comparison test. * *P* < 0.05 was considered as statistically significant.

## Results

### Foxp1 is expressed in RGC, RPE and choroidal endothelial cells

**Fig. 1:**
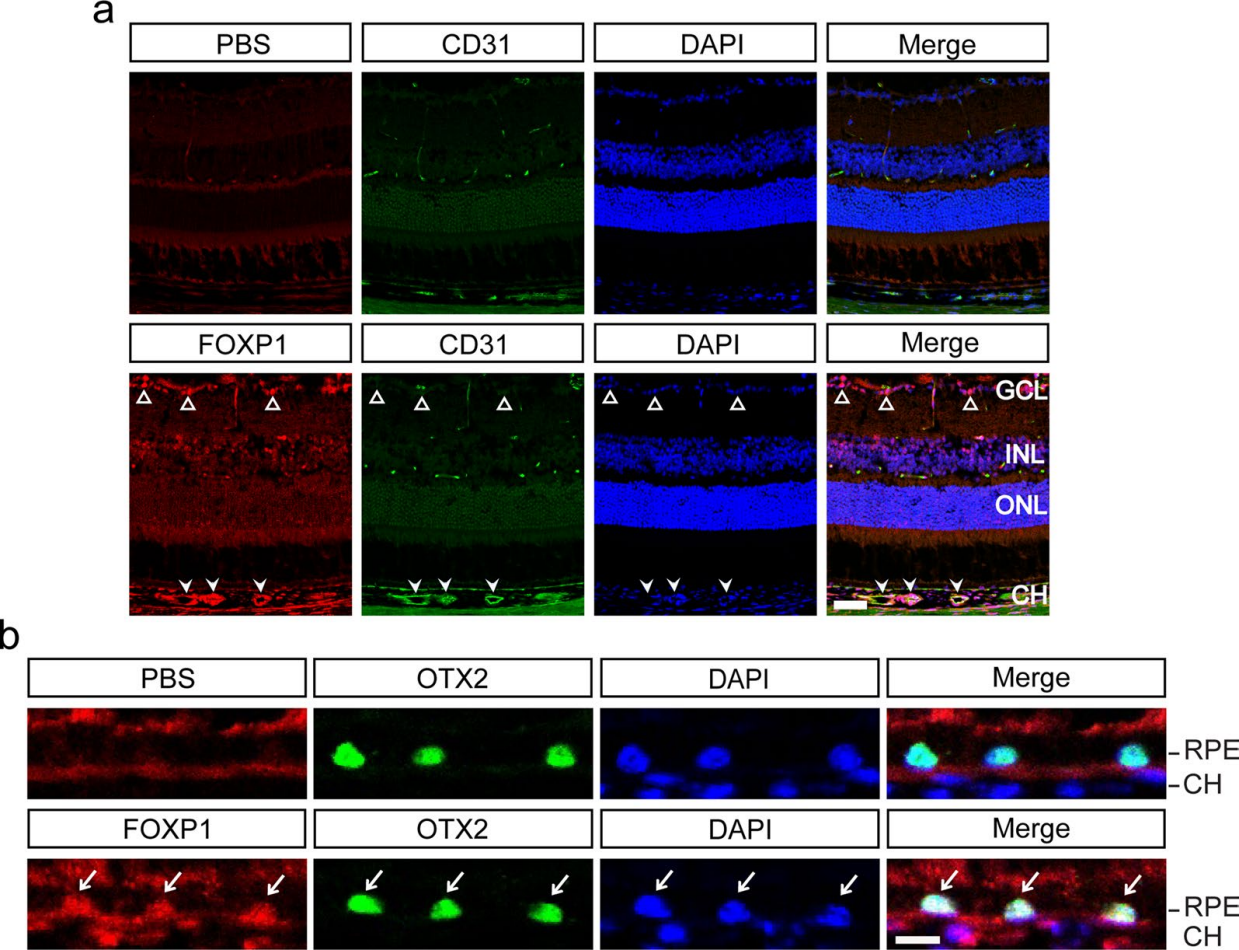
Foxp1 is expressed in the mouse retina-choroid. **a** Co-immunostaining of Foxp1 (red) and CD31 (green) in the eyes of 6 to 8 weeks C57BL/6 J mice. Sections incubated with phosphate buffered solution (PBS) without the primary antibody were used as the negative control. Foxp1 is expressed in retinal ganglion cells (triangles) and is present in the CD31-positive endothelial cells in the choroid (arrowheads). Nuclei were counterstained with DAPI (blue). Scale bar = 50 μm. **b** Foxp1 (red) was colocalized with OTX2 (green), a marker of retinal pigment epithelium cells (arrows). Scale bar = 10 μm. GCL, ganglion cell layer; INL, inner nuclear layer; ONL, outer nuclear layer; RPE, retinal pigment epithelium; CH, choroid

Foxp1 is widely expressed in human and murine tissues. FOXP1 mRNA and protein are found in the lymph node, lungs, brain, kidneys, endocrine organs, and reproductive systems [15]. In the mouse brain, Foxp1 is expressed in the projection neurons of the neocortex and the CA1/CA2 hippocampal subfields [23, 24]. In the neural retina, Foxp1 is partially colocalized with Foxp2 in retinal ganglion cells (F-RGCs) [25]. To delineate the overall pattern of Foxp1 expression in the mouse eye, we performed immunofluorescence staining of Foxp1 on cryostat-sectioned eyeballs. Foxp1 staining was found in the ganglion cell layer, as expected for F-RGCs. Irregular spots of Foxp1 were also noticed in the choroid where they colocalized with CD31-positive endothelial cells (Fig. 1a). It is possible that Foxp1 participates in the formation and maintenance of the choroidal vascular bed. Interestingly, Foxp1 was present in the RPE layer and colocalized with OTX2, a marker for RPE cells (Fig. 1b). RPE cells have been shown to produce VEGF and initiate the process that leads to pathologic neovascularization in AMD [26, 27].

**Fig. 2:**
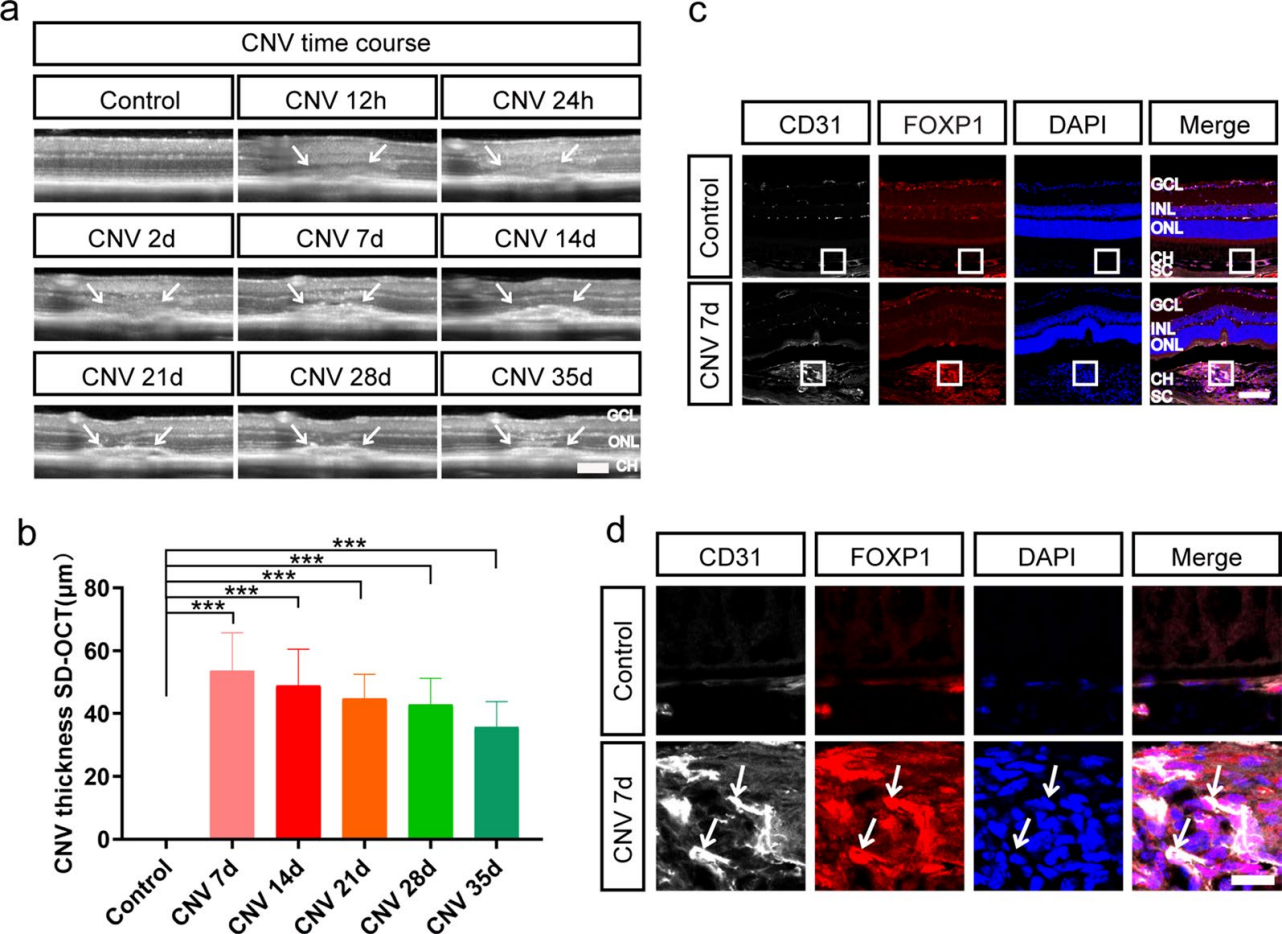
Choroidal neovascularization (CNV) is accompanied by increased Foxp1 expression. **a** Representative spectral domain optical coherence tomography (SD-OCT) B-scan images at the indicated times following laser treatment. Arrows point to the location of CNV in the choroid. Scale bar = 50 μm. **b** CNV thickness was calculated from the SD-OCT images. **c** Cryostat sections from the eyes burned by the laser were immunostained at day 7 with the antibodies against CD31 (white) and Foxp1 (red). Scale bar = 100 μm. **d** Magnified images of the square box in (**c**). Arrows depict the colocalization of Foxp1 and the endothelial marker CD31. Nuclei were counterstained with DAPI (blue). Scale bar = 20 μm. GCL, ganglion cell layer; INL, inner nuclear layer; ONL, outer nuclear layer; CH, choroid; SC: sclera. ***: *P* < 0.001, n = 3 in each group. Statistical analysis was performed by one-way ANOVA followed by Tukey’s post-hoc test

To study whether Foxp1 is involved in the neovascular AMD, we generated CNV in vivo model by laser-induced photocoagulation, a procedure relying on laser injury to perforate Bruch’s membrane. The destruction led to the growth of new blood vessels from the choroid into the subretinal space, which recapitulates the main features of the exudative form of human AMD [28]. CNV was induced in age-matched C57BL/6 J mice that were randomly assigned to control and laser-treatment groups (6 to 8 weeks old). The CNV lesions, imaged by optical coherence tomography [29], exhibited a spindle-shaped hyper-reflective area in the subretinal space (Fig. 2a). The histopathological progression of angiogenesis can be arbitrarily divided into two phases, injury/angiogenesis (days 1 to 6 post-laser) and the mature/repairing phase (after day 7) [30]. Maximum CNV was observed around day 7 after the laser burn, followed by slow regression or tissue repairing that led to the reduction of the CNV thickness (Fig. 2b). The maximal CNV on day 7 enclosed many CD31-positive endothelial cells. Foxp1 was also present in the endothelial cells of the CNV (Fig. 2c–d). As Foxp1 in vitro promotes endothelial cell proliferation, migration, and sprouting [12], an inhibition of Foxp1 in the choroidal endothelium could suppress the development of CNV.

### Six3-Cre deletes Foxp1 in the RPE, but not the endothelial cells, leading to reduced choroidal VEGF

**Fig. 3:**
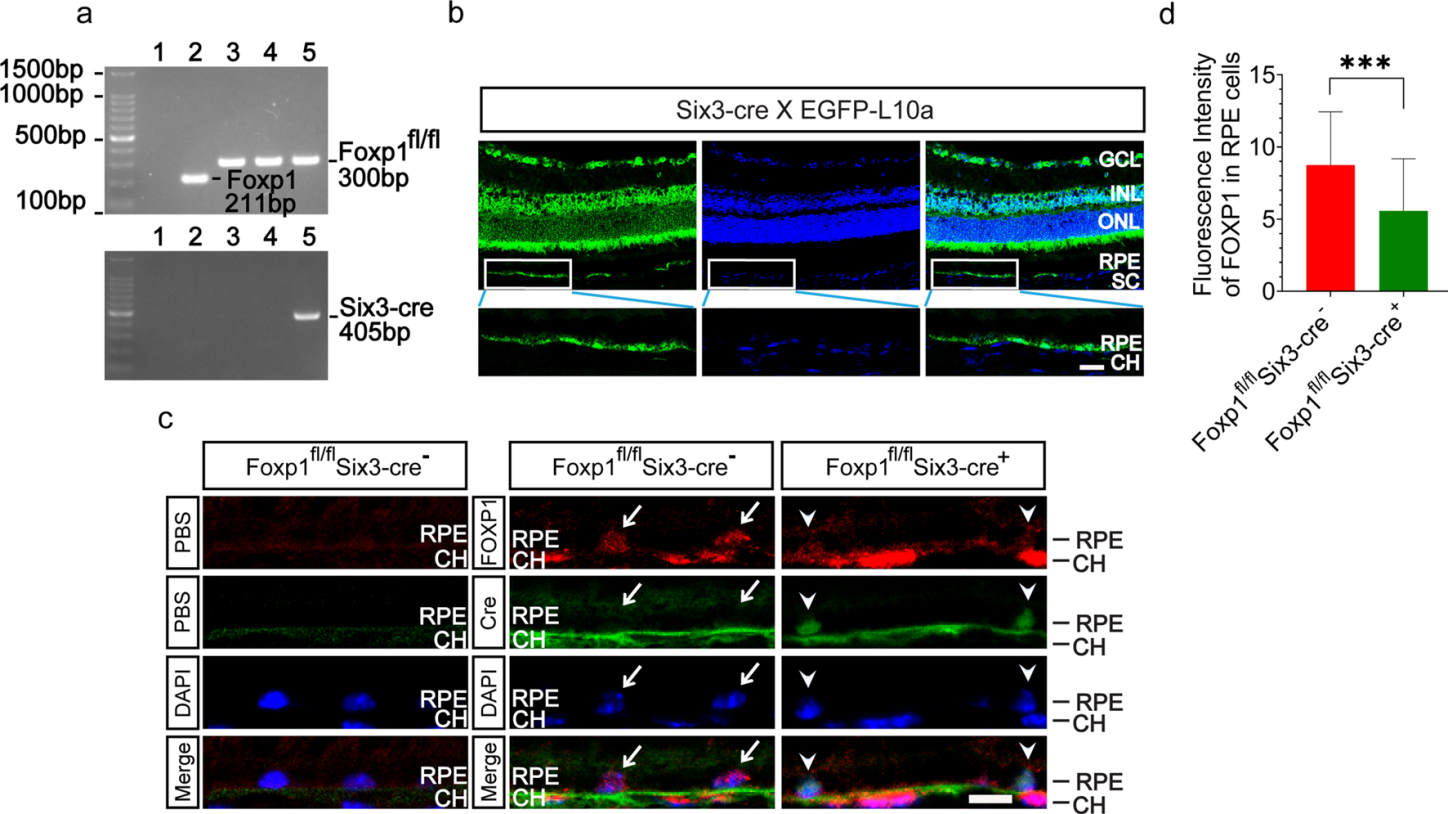
Six3-Cre deletes Foxp1 in the retinal pigment epithelium (RPE) but not endothelial cells. **a** Polymerase chain reaction (PCR) genotyping of the tail-extracted DNA from the knockout (KO) and control mice. Wild-type (WT) Foxp1 allele generated a band of 211 bp, smaller than that from the floxed Foxp1 (Foxp1^fl/fl^) (300 bp). The Foxp1 KO (lane 5) was identified by the presence of floxed Foxp1 and the Cre (405 bp) (Foxp1^fl/fl^Six3-Cre^+^) in the progenies of Foxp1^fl/fl^ and Six3-Cre mice. The littermate control (Foxp1^fl/fl^Six3-Cre^−^) was characterized to harbor the floxed allele of Foxp1 but without Cre. Lane 1: water; Lane 2: WT Foxp1; Lane 3: Foxp1^fl/fl^; Lane 4: Foxp1^fl/fl^Six3-Cre^−^; Lane 5: Foxp1^fl/fl^Six3-Cre^+^. **b** Cryostat sections from the adult Six3-Cre transgenic mice crossed with the EGFP-L10a reporter mouse strain. The enhanced green fluorescence (EGFP) indicates that Cre-mediated recombination occurred in the ganglion cell layer (GCL), inner nuclear layer (INL), and RPE. Nuclei were counterstained with DAPI (blue). Scale bar = 20 μm. **c** Eye sections from the control and Foxp1 KO mice were immunostained with Foxp1 (red), Cre (green), and DAPI (blue). Negative controls were incubated with phosphate buffered solution (PBS) in lieu of the respective primary antibodies. Arrows depict the cells expressing FOXP1 in the RPE in control mice without Six3-Cre. The Foxp1 signal was weaker in the RPE of KO mice (Arrowheads). Scale bar = 10 μm. **d** Quantification of mean fluorescence intensity of Foxp1 in RPE cells. CH, choroid; ***: *P* < 0.001, n = 188 cells for the control and 214 for the Foxp1 KO mice. Statistical comparisons were made using Student’s *t*-test

To assess Foxp1 contribution to the CNV, we generated Foxp1 double deficient mice by crossing Foxp1^flox/flox^ with the Six3-Cre mice. Six3-Cre mice express nuclear-localized Cre recombinase under the control of the murine Six3 promoter, a transcription factor expressed in the retina and the ventral aspect of the eye [31]. The knockout progenies were identified by the expression of floxed Foxp1 (300 bp) and Cre enzyme (405 bp) (Fig. 3a). To determine where precisely the Cre was expressed in the eye, we crossed the Six3-Cre with a reporter mouse strain, EGFP-L10a. Expression of the Cre enzyme drove the removal of a loxp-flanked stop cassette placed between the promoter and the fusion protein of EGFP and ribosomal L10a [21], and thus marked the site of recombination by EGFP. The reporter EGFP was found to be expressed in the GCL (Ganglion cell layer) and INL (Inner nuclear layer) of the retina. Unexpectedly, we found that the RPE was also labeled by fluorescence. However, no EGFP signal was detected underneath the RPE**,** suggesting that Cre recombination occurred in the RPE but not in choroidal endothelial cells (Fig. 3b).

To confirm the Cre expression and the knockout of Foxp1 in RPE cells, we examined the expression of Foxp1 and Cre by immunofluorescence. No immunostaining was present in the control sections that eliminated the first antibody. Foxp1 was detected in the RPE of the progeny that did not express Six3-Cre. In the KO mice with Cre expression, Foxp1 staining was weakened (Fig. 3c–d), indicating that Cre-mediated recombination in the RPE caused excision of the floxed Foxp1.

**Fig. 4:**
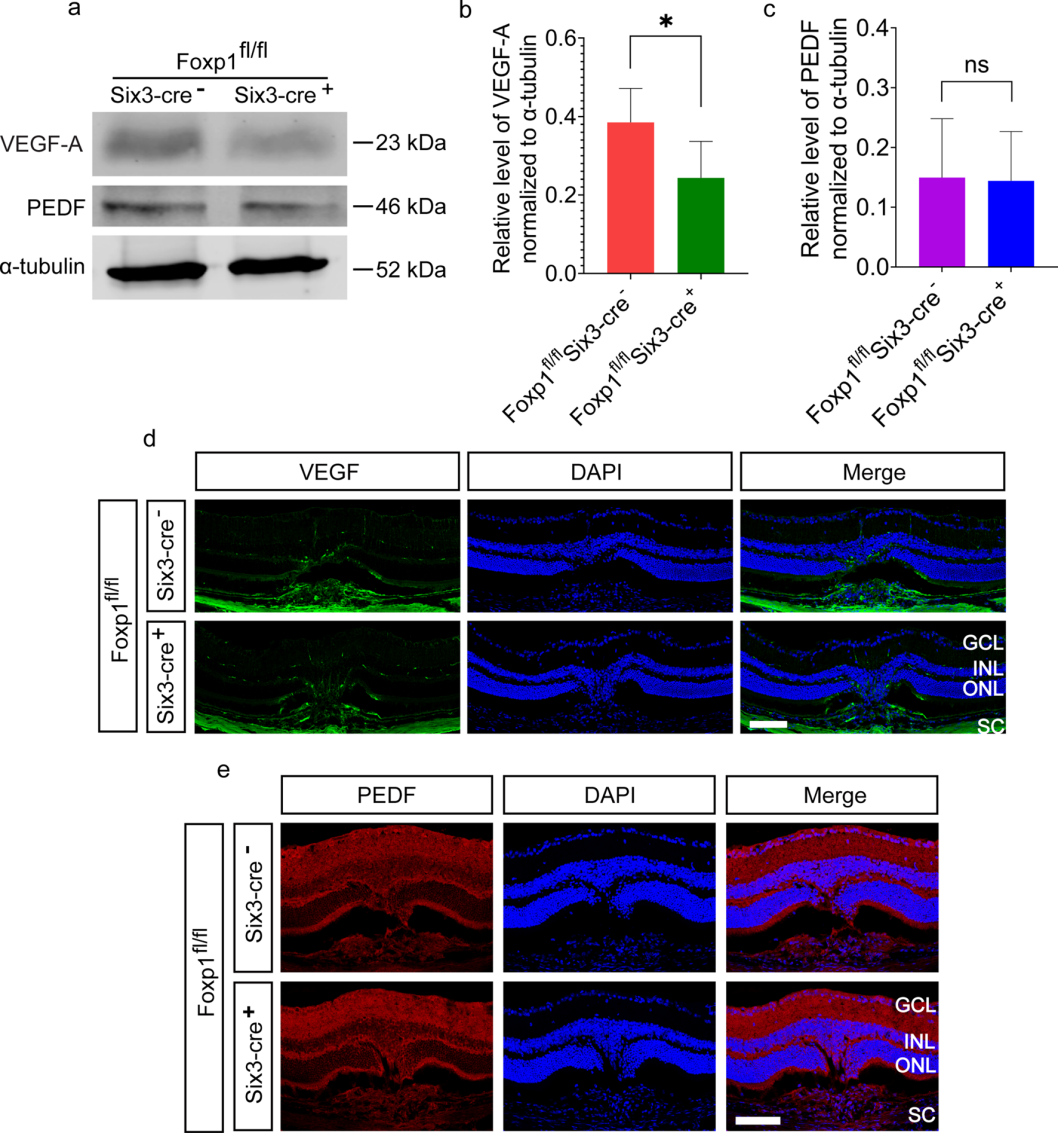
Foxp1 KO reduces vascular endothelial growth factor A (VEGF-A) expression but not pigment epithelium derived factor (PEDF). **a** Representative Western blot of VEGF-A in RPE-choroid lysates of Foxp1-KO (Foxp1^fl/fl^Six3-Cre^+^) and control mice (Foxp1^fl/fl^Six3-Cre^−^). α-tubulin was used as the loading control. VEGF-A protein was lower in Foxp1-KO mice than in the control. **b**–**c** Relative densitometry of VEGF-A and PEDF normalized to α-tubulin. ns: no significant difference. **d**–**e** Immunostaining of VEGF (green) and PEDF (red) in the KO and control from laser-treated mice on day 7. VEGF staining was reduced around choroidal neovascularization (CNV) in the KO mice compared to the control. Scale bar = 100 μm. RPE, retinal pigment epithelium; GCL, ganglion cell layer; INL, inner nuclear layer; ONL, outer nuclear layer; SC, sclera; *: *P* < 0.05, n = 4, Student’s *t*-test

RPE maintains the choriocapillaris (CC) in the normal eye and participates in the pathogenesis of CNV in AMD [32]. RPE-related changes have been found in patients with neovascular age-related macular degeneration [33]. Alterations in RPE-secreted factors are sufficient to cause vascular changes and CNV in an iPSC-RPE-CC model [34]. VEGF is produced by the RPE cells and acts as paracrine signaling between RPE and CC [35]. Blocking VEGF signaling pathway inhibits ocular neovascularization [36]. On the contrary, over-expression of VEGF in the RPE can lead to CNV [2]. All these studies demonstrate that secretion of VEGF from the RPE is a critical determinant for CNV. Therefore, we quantified the expression levels of VEGF-A and PEDF from the isolated RPE-choroid tissue by Western blotting. VEGF-A, but not PEDF, was significantly reduced in Foxp1 KO mice (Fig. 4a–c). Consistently, VEGF immunostaining was weaker in the KO mice than in the control (Fig. 4d–e), suggesting that VEGF is under Foxp1’s regulation in the RPE.

### Foxp1 knockout inhibits laser-induced CNV but does not affect the normal vasculature

**Fig. 5:**
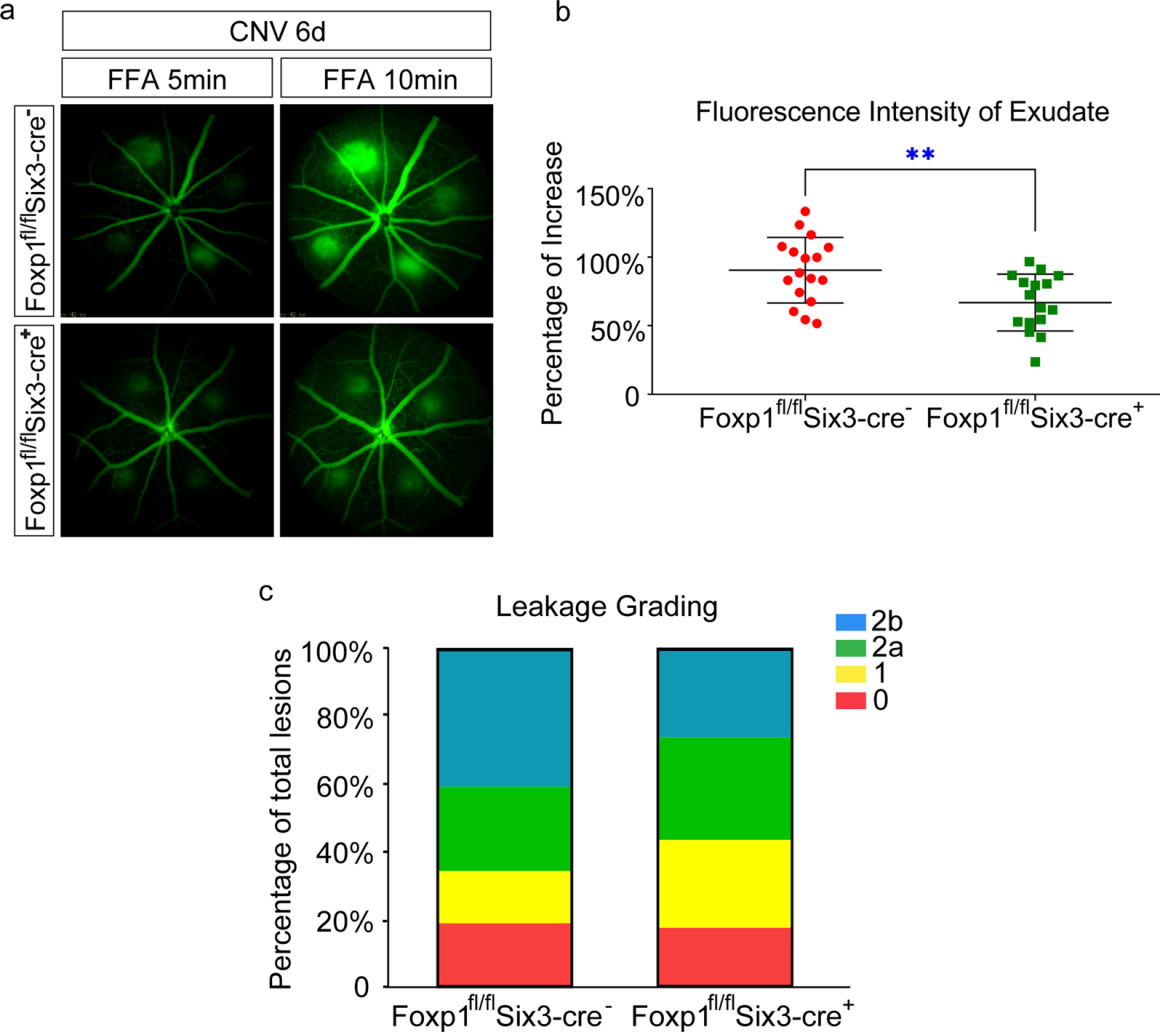
Foxp1 KO reduces leakage of fluorescein sodium in the laser-induced choroidal neovascularization (CNV). **a** Fundus fluorescein angiography (FFA) images in control (Foxp1^fl/fl^Six3-Cre^−^) and KO mice (Foxp1^fl/fl^ Six3-Cre^+^) at 5 and 10 min after intraperitoneal injection of fluorescein sodium at day 6 post-laser injury. Fluorescence in the CNV spots was increased at 10 min from that at 5 min. **b** Percentage of the increase in fluorescence intensity (FI), which was quantified from FFA at 10 over 5 min. **c** The level of leakage was assessed by leakage grading. Grade 0: not leaky; Grade 1: questionable leakage; Grade 2a: leaky; Grade 2b: pathologically significant leakage. The pathological leakage was reduced in the KO mice. **: *P* < 0.01, n = 4, Student’s *t*-test

**Fig. 6:**
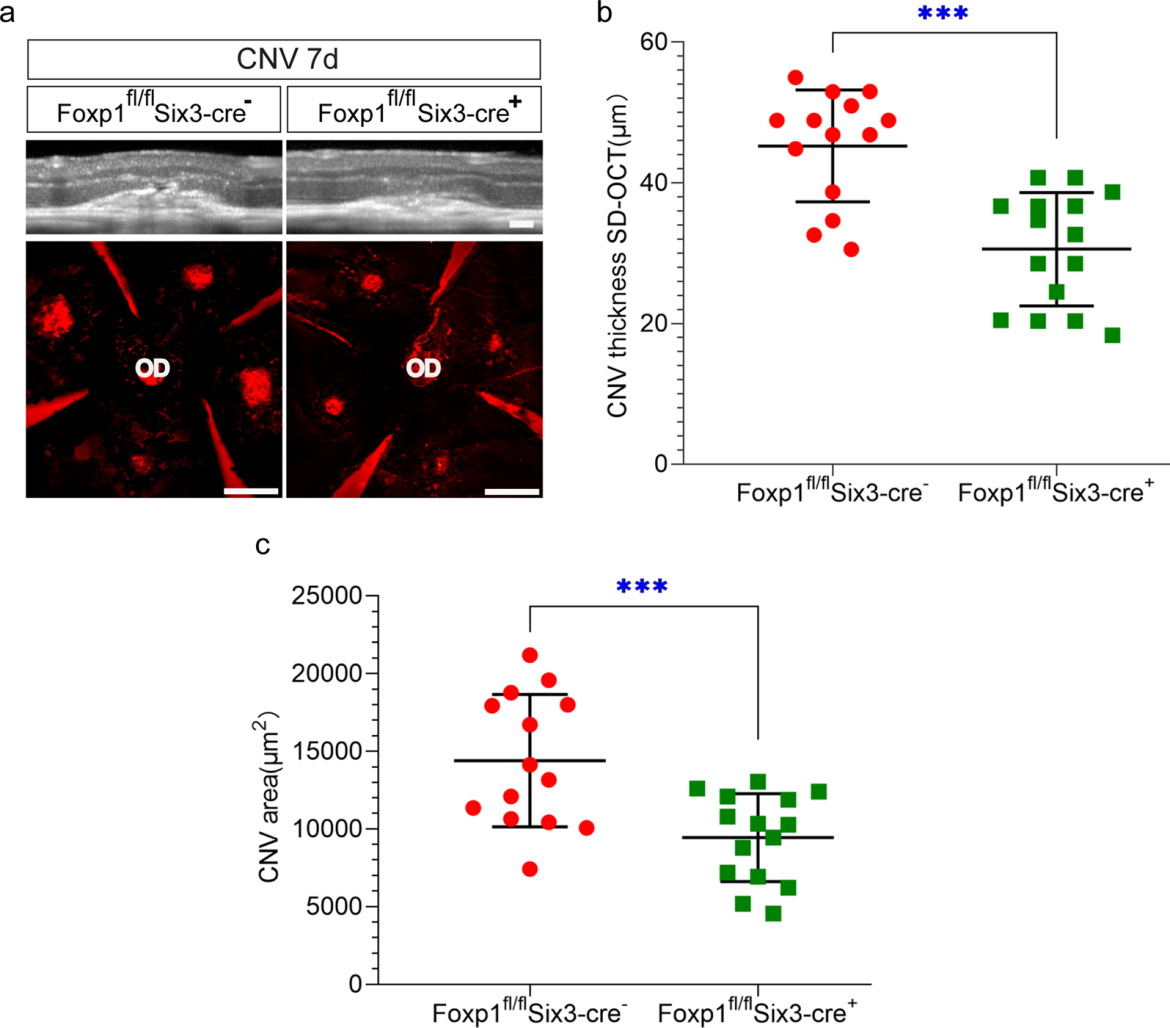
Foxp1 KO partially reduces laser-induced choroidal neovascularization (CNV) lesions. **a** RPE-choroid-sclera complex was dissected from Foxp1-KO mice and the littermate control at day 7 following the laser burn. CNV was examined by spectral domain optical coherence tomography (SD-OCT) scanning and fluorescent B4 Isolectin staining (red). Scale bar 50 μm in SD-OCT and 10 μm in IB4 staining. **b**–**c** CNV lesion thickness and area were calculated from the SD-OCT scanning and the fluorescent images of the CNV, respectively. OD, optic disc; ***: *P* < 0.001, n = 4, Student’s *t*-test

To study the roles of Foxp1 in CNV, we performed laser-induced CNV in control and KO mice. The procedure generated four laser spots per eye centered around the optic disc. Heat coagulation by laser led to inflammation of the choroidal tissue. We examined the inflammation-induced vascular leakage with FFA on day 6. The mice were injected with fluorescein sodium intraperitoneally, and the fluorescence was examined at 5 and 10 min after the injection. Hyper-fluorescence appeared in the laser spots of the wild-type (WT) mice at 5 and 10 min, indicating a high permeability and reduced clearance of fluorescein in the newly formed choroidal vessels. In contrast, the fluorescence from the laser-induced CNV in the KO mice was lower, and the percentage of the increase in FI from 5 to 10 min was reduced by the KO of Foxp1 (WT, 90% *vs.* Foxp1 KO, 68%; Fig. 5a–b). By leakage grading, the CNV lesion with pathologically significant leakage (grade 2b) was reduced in KO mice (Fig. 5c). After recovery from the anesthesia-related lens opacification in FFA, fundus images were captured by SD-OCT on day 7 (post laser injury). The thickness of CNV as measured by OCT scanning decreased from 45.2 to 30.6 μm by Foxp1 KO (Fig. 6a–b). We next analyzed neovessel formation by lectin staining. The eyes were harvested and stripped of the retina. RPE-choroid-sclera flat-mounts were prepared and stained with Alexa Fluor® 594-conjugated GSL I-B4 isolectin. The isolectin-positive area of laser-induced CNV spots in the control was 14386 μm^2^, whereas the average spot in Foxp1 KO mice was 9445 μm^2^ (Fig. 6c), demonstrating that neovascularization was reduced. Together, the data shows that the knockout of Foxp1 dampens vascular leakage and inhibits pathological angiogenesis of CNV in mice.

**Fig. 7:**
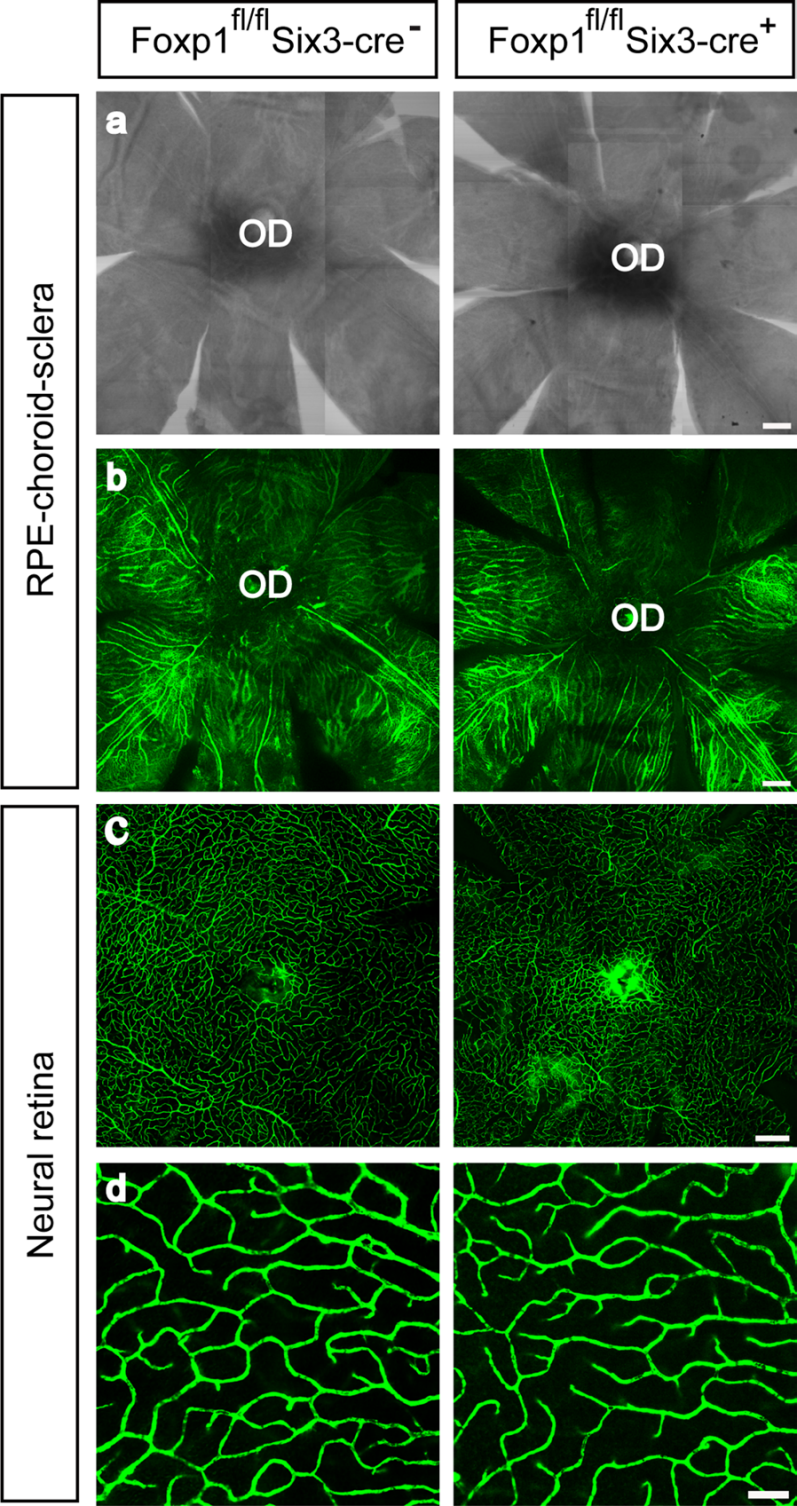
Foxp1 KO does not change the apparent vascular bed. Adult control and Foxp1 KO mice were perfused with fluorescein isothiocyanate dextran (FITC-dextran) through the left ventricle. The melanin bleaching procedure using H_2_O_2_ was applied to the whole eyeballs. The retina and RPE-choroid-sclera complex were separated and flat-mounted on the slides (n = 4 mice). **a** Bright-field views of the RPE-choroid-sclera layers with the pigment bleached. Scale bar = 200 μm. **b** Fluorescent images of the FITC-dextran from the RPE-choroid-sclera complex. No apparent shrunken area or accumulation of fluorescence was detected. OD: optic disc. Scale bar = 200 μm. **c** Fluorescent micrographs of the retinal capillary. Scale bar = 200 μm. **d** High-magnification pictures showing the deep plexus layer of retinal capillaries. Scale bar = 50 μm

VEGF serves a trophic role for choroidal vessels that nourish the outer retinal layers. To determine whether reduced VEGF in Foxp1 KO mice had a profound effect on the vascular bed, we visualized the microvasculature in the choroid and retina. Control and Foxp1 KO mice were injected with FITC-dextran [37]. The neural retina and RPE-choroid-sclera complex from the pigment-bleached eyeballs were flat-mounted and examined by fluorescent microscopy. We did not find any shrunken area or aberrant fluorescent accumulations in the choroid (Fig. 7a–b), suggesting that there was no atrophy or leakage of the choroidal capillaries in the KO mice. Though the KO partially reduced VEGF, the choriocapillaris may only require a base level of VEGF for maintenance. In addition, the central retinal vasculature supplies oxygen and nutrients to the inner retinal layers. Retinal microvascular bed in the KO mice was as normal as that of control; abnormalities such as aneurysm and tuft of neovascular vessels were not detected in the deep plexus layer of the retinal capillary (Fig. 7c–d). These results suggest that Foxp1 in the RPE and retina may not be essential for maintaining blood vessels in the eyes of adult mice.

## Discussion

In this study, we found that knockout of Foxp1 reduces laser-induced CNV in mice. The Six3-Cre did not remove Foxp1 in the choroidal endothelial cells but excised the gene in the retina and RPE, resulting in reduced VEGF in the RPE and choroid and partial reduction of CNV. Since endothelial cell proliferation, migration, and sprouting in vitro depend on Foxp1 [12], blocking Foxp1 in the endothelial cells would also inhibit the growth of new blood vessels. Thus, inhibition of Foxp1 may potentially be a novel strategy for preventing and treating wet AMD. Molecular strategies such as siRNA or CRISPR are available to knockdown or knockout Foxp1 in vivo [24].

Homozygous deletion of Foxp1 is embryonically lethal due to a cardiac defect [38]. Therefore, we took an eye-specific deletion of Foxp1 by crossing Foxp1^flox/flox^ with Six3-Cre mice. The Six3-Cre mice have been used to knock out genes in non-vascular tissues of the eye. It was reported that the Cre is not expressed in endothelial cells and the RPE in developing eyes of E14.5-P5 mice [39, 40]. By crossing with a Cre reporter strain of mice, we confirmed that Six3-Cre was not expressed in the choroidal endothelium. However, we discovered a robust expression of the EGFP reporter in the RPE of adult mice (Fig. 3b), revealing the location of Cre-mediated recombination in the RPE. Considering the aforementioned studies, we speculate that the Six3-Cre may be expressed only in the adult but not developing RPE. A regionally and temporally restricted expression of Cre in the adult RPE would provide a valuable tool for the study of AMD.

RPE produces and secretes soluble VEGF isoforms toward the basolateral side, while the CC preferentially expresses its receptors on the side facing the RPE. The soluble VEGF diffuses across Bruch’s membrane to reach the CC [41], and thus maintains the CC. Abnormal secretion of VEGF promotes the pathogenesis of choroidal neovascularization in AMD [32]. RPE increases the VEGF secretion during the development of AMD upon noxious stimulations, which include hypoxia, oxidative stress, hyperglycemia, and cytokines [42]. It is unclear how VEGF may be regulated by the loss of Foxp1 in RPE cells. However, in murine bone marrow-derived dendritic cells, silencing Foxp1 inhibits the production and secretion of inflammatory cytokines such as TNF-α, IL-1β, and IL-6. Conversely, overexpression of Foxp1 increases cytokine production [43]. Knockout of Foxp1 in RPE might repress the cytokines, thereby reducing the stimulation for the production or secretion of VEGF in response to the laser-photocoagulation.

RPE is also one of the sources that provide VEGF during the development of diabetic retinopathy [44]. Diabetic retinopathy is a common complication of diabetes mellitus that causes retinal neovascularization, and in the late-stage of the disease, results in macular edema and vision loss. VEGF is a compelling therapeutic target for diabetic retinopathy [45]. We do not know whether VEGF in other retinal cells may be regulated by Foxp1 as well. Nevertheless, the protective physiological and vision-threatening pathological functions played by VEGF render the regulation of VEGF a highly interesting topic for future exploration.

## Conclusions

We showed that transcription factor Foxp1 is expressed in RGC, RPE, and the choroidal endothelial cells of mice. Six3-Cre-mediated recombination of Foxp1^flox/flox^ knockouts the Foxp1 in RPE, leading to reduced VEGF in RPE-choroid tissue. The knockout of Foxp1 inhibits laser-induced CNV in mice. The results suggest that Foxp1 may be essential for the induction of CNV. Foxp1 could potentially be a novel target for prevention of and therapy for neovascularization related to exudative AMD.

## Data Availability

All data generated or analyzed during this study are included in this text.
